# Interactive Effects of Shading Intensity and Container Type on Growth, Photosynthesis, and the Carbon–Antioxidant Trade-Off in *Dalbergia odorifera* Seedlings

**DOI:** 10.3390/plants15203242

**Published:** 2026-10-21

**Authors:** Mengwen Zhang, Feifei Chen, Ling Lin, Lin Chen, Xiaoli Yang, Xiaona Dong, Jiaming Song, Chuanteng Huang

**Affiliations:** 1 Hainan Academy of Forestry (Hainan Academy of Mangrove), Haikou 571100, China; zaizai_qq@163.com (M.Z.); cfifi@163.com (F.C.); lling008@163.com (L.L.); 13707569339@163.com (L.C.); 18889827255@163.com (X.Y.); dongxiaona@163.com (X.D.); 15035584294@163.com (J.S.); 2 The Innovation Platform for Academicians of Hainan Province, Haikou 571100, China; 3 Key Laboratory of Tropical Forestry Resources Monitoring and Application of Hainan Province, Haikou 571100, China

**Keywords:** antioxidant defense, non-woven fabric containers, photosynthetic physiology, resource allocation, shading intensity

## Abstract

*Dalbergia odorifera* T. Chen is a rare and endangered tree species endemic to China. However, efficient cultivation protocols for high-quality seedling production remain to be optimized. This study investigated the interactive effects of shading intensity and container type on seedling growth, photosynthesis, antioxidant systems, and resource allocation. A two-factor completely randomized design was used with five shading levels (0%, 20%, 40%, 60%, and 80%) and three container types (rigid plastic pots, non-woven fabric containers, and black plastic bags). Shading suppressed growth by limiting carbon assimilation, with total dry weight reduced by 93–94% under heavy shade, triggering a carbon–antioxidant trade-off. Moderate shading (40–60%) increased antioxidant enzyme activities, but 80% shading caused antioxidant dysfunction and membrane lipid peroxidation, with malondialdehyde (MDA) content increasing by 343%. Non-woven fabric containers improved root development and maintained higher photosynthetic efficiency under shade, whereas black plastic bags exacerbated stress. Container type significantly interacted with shading intensity: non-woven fabric containers performed best under 0–20% shading, while rigid plastic pots showed advantages under 60–80% shading. Principal component analysis identified full sunlight combined with non-woven fabric containers as the optimal cultivation model. These findings suggest that container type modulates light responses, potentially through changes in the rhizosphere environment, and offer practical guidance for precision cultivation of *D. odorifera* and other valuable tree species.

## 1. Introduction

*Dalbergia odorifera* T. Chen, an evergreen tree species of the Fabaceae family endemic to China, is currently classified as endangered [1,2]. Valued for its superior timber quality and medicinal heartwood, this species holds considerable economic and ecological significance [3,4]. However, its sustainable utilization is severely constrained by poor natural regeneration and critically depleted wild resources, necessitating the development of efficient plantation cultivation [5]. Seedling quality is fundamental to successful plantation establishment [6]. Among the manageable factors in nursery production, the light environment and container type critically determine seedling morphogenesis and physio-ecological adaptability [7]. Therefore, elucidating the interactive regulation of light intensity and container type is a theoretical prerequisite for advancing precision nursery cultivation of this valuable species.

Light is the primary environmental factor governing seedling morphogenesis and photosynthetic carbon metabolism [8]. It provides the energy for photosynthesis and regulates plant growth, development, and metabolic pathways [9]. Plants have evolved adaptive strategies to cope with light fluctuations: under high light, they mitigate photodamage through stomatal regulation and non-photochemical quenching [10,11]; under low light, they increase pigment biosynthesis and optimize photosystem II efficiency [12,13]. These adjustments can be assessed non-destructively via chlorophyll fluorescence [14,15]. In nursery production, moderate shading enhances light-use efficiency and promotes carbon assimilation and biomass accumulation [16], whereas excessive shading or full sunlight in summer can cause photoinhibition and photooxidative stress, impairing carbon fixation and seedling development [17,18,19]. The seedling stage is particularly sensitive, and light adaptation is critical for survival and population establishment [20,21]; thus, understanding these mechanisms is ecologically and practically important. However, the optimal light intensity varies among species: 30–50% shading benefits *Corylus chinensis* Franch. [22], *Sorbus tianschanica* Rupr. [23], and *Photinia magnoliifolia* Z.H. Chen [24], while *Keteleeria fortunei* (A. Murray bis) Carrière [25] and *Cyclobalanopsis gilva* (Blume) Oerst. Reference [26] performs best at 60% and 50% shading, respectively. These differences reflect variations in light compensation points, saturation points, and photosynthetic enzyme efficiency [27]. Notably, even light-demanding species show complex responses [28], because light not only drives carbon assimilation but also influences carbon allocation between growth and defense [22]. When light exceeds the optimal range, photoprotection increases energy consumption even without photoinhibition, limiting carbon for growth and antioxidant defense [9]. This allocation trade-off may be modulated by the rhizosphere environment, as root water and nutrient uptake affect shoot photosynthesis and stress tolerance [12,29]. Consequently, light intensity effects may not be independent of cultivation containers but interact with the container-created rhizosphere microenvironment.

Container type, as a fundamental determinant of the rhizosphere microenvironment, profoundly influences seedling quality by modulating root architecture and physiological function [30,31]. Among the three container types evaluated in this study, rigid plastic pots are widely used in nursery production due to their durability and reusability, but their rigid walls can restrict root expansion and may lead to root circling if drainage or aeration is inadequate [32]. Non-woven fabric containers are characterized by high air permeability and root penetration capability; they effectively prevent root circling, promote fibrous root development, and shorten post-transplant acclimatization [32,33]. Moreover, non-woven containers significantly increase fine root density and induce divergent root distribution patterns [33]. Black plastic seedling bags are the most widely used disposable containers in tropical and subtropical nurseries because of their low cost and excellent moisture retention, but their poor aeration can lead to CO_2_ accumulation, O_2_ depletion, and elevated root-zone temperatures [34,35]. Nevertheless, black plastic containers may promote biomass accumulation and chlorophyll synthesis in young seedlings due to their thermal and moisture retention properties [36,37]. For *D. odorifera*, preliminary investigations suggest that non-woven fabric containers outperform rigid plastic pots in promoting root expansion, while black plastic containers may confer advantages in enhancing chlorophyll content and photosynthetic rates [38,39]. However, existing studies have predominantly focused on evaluating the effects of individual container types in isolation, with systematic comparative analyses across container types remaining scarce.

The interaction between light intensity and container type is a critical scientific question in container seedling cultivation research [40]. Seedling growth depends on the coordination of above-ground light capture and below-ground resource acquisition [41]; this coordination is particularly relevant when considering how container-mediated rhizosphere conditions modulate light responses. Existing studies have confirmed significant interactive effects between paired factors on seedling photosynthetic physiology, such as light and nitrogen [42,43] or light and nutrient ratios [44,45]. These findings indicate that the regulatory influence of a single factor often depends on the state of other environmental variables. In nursery production, light intensity and container type represent two readily manipulable factors. However, their potential interactions have been rarely examined systematically [5]. Containers may profoundly influence seedling perception of and response thresholds to light intensity signals by modulating rhizosphere aeration, thermal conditions, and root architecture [30]. For *D. odorifera*, experimental evidence and mechanistic understanding are lacking regarding whether light and container type interact, and how such interactions regulate seedling phenotypes, ranging from physiological processes (e.g., photosynthesis, antioxidant balance) to morphological traits (e.g., biomass allocation, root–shoot coordination) [7]. Addressing this gap is essential for moving beyond empirical practices and developing refined, physiology-based cultivation protocols.

Therefore, this study was designed to investigate the interactive effects of shading intensity and container type on the growth and physiological responses of *D. odorifera* seedlings. We hypothesized that (i) shading intensity and container type would interactively affect seedling growth, photosynthetic performance, and antioxidant responses; (ii) shading would suppress growth by limiting photosynthetic carbon assimilation, leading to a trade-off between carbon gain and antioxidant defense; and (iii) non-woven fabric containers would generally outperform the other container types, whereas black plastic bags would perform poorly. To test these hypotheses, we evaluated photosynthetic capacity, antioxidant defense, and morphological plasticity in response to these factors. The findings are expected to provide a theoretical foundation and practical parameters for precision cultivation of high-quality *D. odorifera* seedlings, as well as a methodological reference for stress physiology and intensive nursery production in other rare leguminous or broadleaved tree species.

## 2. Results

### 2.1. Effects of Shading Intensity and Container Type on Seedling Growth and Biomass Accumulation

Two-way ANOVA revealed that both shading intensity and container type had highly significant (*p* < 0.01) main effects on plant height increment, basal diameter increment, root dry weight, leaf dry weight, and total dry weight. The shading intensity × container type interaction was highly significant (*p* < 0.01) for basal diameter increment and total dry weight, significant (*p* < 0.05) for plant height increment and leaf dry weight, but not significant for root dry weight. In contrast, stem dry weight was significantly affected only by shading intensity (*p* < 0.01), with no significant influence from container type or their interaction (*p* > 0.05) (Table 1).

Within the same container type, plant height increment exhibited distinct response patterns to increasing shading intensity. In rigid plastic pots and black plastic bags, it increased initially and then declined, with the highest value at 20% shading. In non-woven fabric containers, it decreased initially and then increased, reaching a minimum at 60% shading. Under the same shading intensity, non-woven fabric containers had significantly higher plant height increment than the other two container types at 0%, 20%, and 80% shading, whereas at 40% and 60% shading, the highest values were observed in black plastic bags and rigid plastic pots, respectively (Figure 1A).

Basal diameter increment declined monotonically with increasing shading intensity, with reductions of 45%, 54%, and 47% in rigid plastic pots, black plastic bags, and non-woven fabric containers, respectively. At each shading level, seedlings in non-woven fabric containers exhibited significantly greater basal diameter increment than those in the other two container types (Figure 1B).

Biomass accumulation decreased consistently with increasing shading intensity. At 80% shading, compared with full sunlight, root dry weight decreased by 92%, 79%, and 81% in rigid plastic pots, non-woven fabric containers, and black plastic bags, respectively; total dry weight decreased by 93%, 92%, and 94%, respectively. Stem and leaf dry weight reductions ranged from 91% to 93% and 97% to 99%, respectively, across container types. Non-woven fabric containers generally had the highest root, leaf, and total dry weights across shading levels, whereas black plastic bags generally had the lowest values; detailed statistical differences are indicated by letter groupings in Figure 1C–F. Detailed means and standard deviations for plant height increment, basal diameter increment, and organ-specific biomass across all 15 treatment combinations are provided in Table A1.

### 2.2. Synergistic Regulation of Photosynthetic Physiology by Shading Intensity and Container Type

Two-way ANOVA revealed that shading intensity, container type, and their interaction exerted highly significant effects (*p* < 0.01) on *P*_n_, *T*_r_, and *C*_i_. Tchl was significantly influenced by shading intensity and the shading × container interaction (*p* < 0.01), but not by container type alone (*p* > 0.05). *g*_s_ was significantly affected only by shading intensity (*p* < 0.01), with no significant main effect of container type or interactive effect (Table 2).

Within each container type, *P*_n_, *T*_r_, and *g*_s_ declined significantly with increasing shading intensity, with reductions of 68–87%, 38–57%, and 66–70%, respectively (Figure 2A–C). Concurrently, *C*_i_ exhibited a marked increase, rising by 123–170% (Figure 2D). Tchl responded differently among container types: it increased continuously in non-woven fabric containers and black plastic bags (by 115% and 79%, respectively), whereas in rigid plastic pots it increased initially and then declined, peaking at 40% shading (Figure 2E).

Across shading treatments, non-woven fabric containers generally had the highest *P*_n_ values, whereas black plastic bags generally had the lowest (Figure 2A). Container differences in *T*_r_, *g*_s_, and *C*_i_ varied with shading intensity; overall, non-woven fabric containers showed the highest *T*_r_ and *g*_s_ under 40–80% shading, whereas black plastic bags showed the lowest *P*_n_, *T*_r_, and *g*_s_ under most shading levels (Figure 2B–D). For Tchl, rigid plastic pots had the highest values under 0–40% shading, while non-woven fabric containers had the highest values under 60–80% shading (Figure 2E). Detailed statistical differences among container types and shading levels are indicated by letter groupings in Figure 2. The complete datasets for photosynthetic parameters and total chlorophyll content across all treatment combinations are presented in Table A2.

### 2.3. Regulation of Antioxidant Systems and Membrane Lipid Peroxidation by Shading Intensity and Container Type

Two-way ANOVA revealed that both shading intensity and container type exerted highly significant main effects (*p* < 0.01) on SOD activity, POD activity, PRO content, and MDA content in seedlings. Except for POD, all other indicators were significantly influenced by the interaction between the two factors (*p* < 0.01) (Table 3).

Within each container type, SOD activity exhibited a single-peak response to increasing shading intensity, increasing initially and then declining, with the highest value at 60% shading (Figure 3A). POD activity followed a similar trend; seedlings in rigid plastic pots and non-woven fabric containers reached maximum POD activity at 60% shading, whereas those in black plastic bags peaked earlier at 40% shading (Figure 3B). The response of PRO content to shading varied by container type: in rigid plastic pots, it increased initially, then decreased, and increased again; in non-woven fabric containers and black plastic bags, it decreased initially, then increased, and decreased again (Figure 3C). MDA content increased with increasing shading intensity across all container types, with overall increases ranging from 230% to 343% at 80% shading compared with full sunlight (Figure 3D).

Under identical shading intensities, the relative rankings of SOD and POD activities among container types were complex and variable. Notably, seedlings in black plastic bags consistently exhibited significantly higher POD activity than the other two container types across all shading treatments, while also generally displaying significantly higher MDA content (with the exception of the 20% shading treatment). Detailed statistical differences among container types and shading levels are indicated by letter groupings in Figure 3. Detailed means and standard deviations for SOD activity, POD activity, PRO content, and MDA content across all treatment combinations are listed in Table A2.

### 2.4. Effects of Shading Intensity and Container Type on Seedling Morphological Structure and Quality Index

Two-way ANOVA revealed that shading intensity significantly affected height-to-diameter ratio, root-to-shoot ratio, and Dickson quality index (*p* < 0.01). Container type exerted a significant effect only on root-to-shoot ratio, while the interaction between shading intensity and container type significantly influenced both height-to-diameter ratio and root-to-shoot ratio (Table 4).

Across all container types, height-to-diameter ratio exhibited a single-peak response to increasing shading intensity, peaking at 20% shading (Figure 4A). The response of root-to-shoot ratio varied by container type: seedlings in rigid plastic pots and non-woven fabric containers reached maximum values at 60% shading, whereas those in black plastic bags showed fluctuations without a clear trend (Figure 4B). Dickson quality index declined continuously with increasing shading intensity, with reductions ranging from 87% to 90% under the highest shading level compared to full sunlight across all container types (Figure 4C).

Under equivalent shading intensities, container-specific differences were evident. For height-to-diameter ratio, the optimal container type varied with shading level: non-woven fabric containers yielded the highest values under 0% and 80% shading; rigid plastic pots performed best under 20% and 60% shading; and black plastic bags peaked under 40% shading (Figure 4A). For root-to-shoot ratio, non-woven fabric containers consistently exhibited the highest values across all shading levels except 0%, where rigid plastic pots were superior, while black plastic bags consistently recorded the lowest values (Figure 4B). Dickson quality index also showed numerical differences among container types across shading intensities: rigid plastic pots performed best under 0% shading, whereas non-woven fabric containers excelled under 20%, 60%, and 80% shading (Figure 4C). Detailed means and standard deviations for the height-to-diameter ratio, root-to-shoot ratio, and Dickson quality index across all treatments are provided in Table A3.

### 2.5. Screening of Best-Performing Treatment Combinations Based on Multivariate Analysis

#### 2.5.1. Interaction Network of Growth-Photosynthesis-Antioxidant Indicators

Correlation analysis revealed that seedling growth parameters (including basal diameter, plant height, and organ-specific dry weights) exhibited highly significant positive correlations (*p* < 0.01) with photosynthetic traits such as *P*_n_, *g*_s_, and *T*_r_, indicating that carbon assimilation capacity directly drives biomass accumulation (Figure 5). Most growth and photosynthetic indicators showed significant negative correlations with *C*_i_, Tchl, POD activity, and MDA content, although the strength and significance level of these correlations varied among parameters. The negative correlation between *P*_n_ and MDA content was particularly pronounced (*r* = −0.79, *p* < 0.01), revealing a tight coupling between carbon assimilation capacity and membrane system integrity.

Notably, a coordinated response was evident within the antioxidant system: SOD activity exhibited highly significant positive correlations with both POD activity (*r* = 0.77, *p* < 0.01) and PRO content (*r* = 0.67, *p* < 0.01), collectively forming an integrated enzymatic and non-enzymatic defense system for reactive oxygen species scavenging. However, both SOD and POD activities were positively correlated with MDA content (*r* = 0.63 and *r* = 0.74, respectively, *p* < 0.05 and *p* < 0.01), suggesting that although the antioxidant system was activated under shading stress, it was insufficient to fully prevent membrane lipid peroxidation. Concurrently, the significant negative correlations between *P*_n_ and both SOD and POD activities (*r* = −0.60 and *r* = −0.77, respectively)) may reflect a trade-off between carbon assimilation and antioxidant defense: under high photosynthetic productivity, resources are preferentially allocated to growth, whereas under low-light stress, a greater proportion of energy must be redirected toward stress defense mechanisms.

#### 2.5.2. Comprehensive Evaluation of Cultivation Effects Based on Principal Component Analysis

Principal component analysis (PCA) was performed on 14 standardized growth and physiological indicators to comprehensively assess the effects of different treatment combinations. The results showed that the first two principal components extracted from the dataset explained 87.80% of the total variance, representing the majority of information from the original variables (Figure 6A). The first principal component (PC1) had an eigenvalue of 10.60 and explained 75.20% of the variance, with high loadings on growth and photosynthetic parameters (including basal diameter, plant height, organ-specific dry weights, *P*_n_, *g*_s_, and *T*_r_). The second principal component (PC2) had an eigenvalue of 1.76 and explained 12.60% of the variance, and was primarily associated with antioxidant system-related indicators such as SOD, POD, and PRO (Figure 6B).

Comprehensive scores for each treatment combination were calculated using the following formula: Comprehensive score = PC1 × 75.20% + PC2 × 12.60%, where PC1 and PC2 are the factor scores for each treatment combination, and 75.20% and 12.60% are the proportions of variance explained by PC1 and PC2, respectively. Significant differences were observed among treatments combining varying shading intensities and container types. The treatment combining 0% shading with non-woven fabric containers achieved the highest comprehensive score (4.13), ranking first, followed by 20% shading with non-woven fabric containers (3.06) and 0% shading with rigid plastic pots (3.01). Comprehensive scores for other treatments progressively declined with increasing shading intensity. Across all shading levels, black plastic bags consistently yielded lower scores than rigid plastic pots and non-woven fabric containers (Figure 6C).

## 3. Discussion

Light intensity is a key environmental factor influencing forest seedling growth and development, with light adaptation strategies varying considerably among tree species [46]. Based on their response patterns to shading, woody plants are broadly categorized as photophilous (optimal growth under full light), shade-tolerant (strong survival under low light), or photophilous with phenotypic plasticity (moderate shading promotes height growth but inhibits biomass accumulation) [9]. In this study, *Dalbergia odorifera* T. C. Chen exhibited a typical photophilous response: basal diameter increment, organ-specific dry weights, and total biomass declined continuously with increasing shading [28]. However, height increment transiently peaked under 20% shading in rigid plastic pots and black plastic bags, revealing a decoupling between height growth and dry matter accumulation. This pattern contrasts with the moderate shade-promoting strategy in *Corylus chinensis* Franch. [22] and *Sorbus tianschanica* Rupr. [23], which achieve synchronous increases in height, biomass, and photosynthesis under 30–50% shading. It is important to distinguish this response from etiolation (skotomorphogenesis), which occurs in complete darkness and is sustained by stored reserves rather than ongoing photosynthesis [47]. In our study, shading was partial (20–80%), and photosynthesis continued, albeit at reduced rates. The transient height peak at 20% shading therefore reflects a shade avoidance strategy under mild light limitation, whereas the decline at higher shading reflects carbon limitation that constrained further elongation [48]. This distinction is further supported by the concurrent reduction in biomass and the absence of continuous stem elongation under heavy shade. Photosynthetically, *P*_n_ and *g*_s_ decreased while *C*_i_ increased with shading, suggesting that non-stomatal factors, rather than CO_2_ shortage, may have contributed to the reduction in *P*_n_ [28]. This aligns with *Ulmus elongata* L. K. Fu & C. S. Ding [29] and *Photinia magnoliifolia* Z.H. Chen [24] but contrasts with *Garcinia paucinervis* Chun ex F. C. How, which maintains photosynthesis under low light via enhanced apparent quantum efficiency [49]. Interspecific differences may lie in PSII sensitivity: in strongly photophilous species, PSII is more sensitive to light fluctuations, and a decline in photochemical quenching under low light may generate excess excitation energy and trigger photoinhibition [50], whereas shade-tolerant species optimize PSII efficiency and light energy allocation [51]. Although container type had no significant effect on *g*_s_, it significantly affected *P*_n_, and its interaction with shading was highly significant; this pattern is consistent with a possible role of non-stomatal factors. Tchl increased with shading in non-woven fabric containers and black plastic bags, and initially increased then decreased in rigid plastic pots. This increase represents a typical acclimation to low light: plants enhance chlorophyll biosynthesis to improve light capture efficiency under reduced irradiance [12]. However, this compensatory response was insufficient to reverse the decline in photosynthetic efficiency. The concurrent rise in intercellular CO_2_ concentration indicates that CO_2_ supply was not limiting; rather, the reduction in *P*_n_ was associated with non-stomatal factors, such as impaired mesophyll photosynthetic activity [13]. Thus, although shading enhanced chlorophyll content, it did not translate into greater carbon assimilation.

The sustained reduction in photosynthetic products triggered cascading effects on the antioxidant system [52]. SOD and POD activities peaked under 40–60% shading, whereas MDA increased continuously, suggesting a potential activation–exhaustion dynamic that is consistent with the resource allocation framework of Monson et al. [53]. The continuous increase in MDA indicates that shading induced progressive oxidative stress: under low light, excess excitation energy that cannot be used for carbon assimilation generates reactive oxygen species (ROS), which attack membrane lipids and lead to lipid peroxidation [47]. This process intensified with shading severity. Correlation analysis further showed that both SOD and POD activities were positively correlated with MDA content (*r* = 0.626 and *r* = 0.740, respectively), indicating that the antioxidant system was activated but insufficient to fully prevent membrane lipid peroxidation. In addition to enzymatic antioxidants, PRO accumulated under shading and appeared to contribute to the antioxidant defense. PRO functions as both a compatible osmolyte and a ROS scavenger: it stabilizes cellular structures, buffers redox potential, and directly detoxifies hydroxyl radicals and singlet oxygen [54,55]. In our study, PRO content increased in rigid plastic pots with shading, and showed complex patterns in other containers, suggesting that PRO accumulation depends on container type and stress severity. However, since PRO was measured as a terminal biochemical endpoint, its specific contribution to mitigating oxidative damage remains inferential and requires direct validation. Under moderate shading (40–60%), seedlings allocated carbon to sustain antioxidant enzyme synthesis and ROS scavenging, keeping membrane damage within limits. At 80% shading, severe carbon limitation (total dry weight reduction 93–94%) was accompanied by a decline in antioxidant enzyme activities and a further increase in MDA, indicating that the antioxidant system may have become insufficient to prevent oxidative damage [56]. However, because ROS were not directly measured, these interpretations remain inferential. Similar trends appear in *Quercus mongolica* Fisch. ex Ledeb., [57] and *Firmiana kwangsiensis* H. H. Hsue [32]. Peak SOD and POD at 40–60% shading, with MDA still rising at 80%, suggest that *D. odorifera* may have a lower antioxidant defense threshold, consistent with its strong photophilous nature: even under low light, excess excitation energy may accumulate until the defense system becomes insufficient to counteract oxidative damage.

Container type profoundly influenced seedling performance by shaping the root system, the primary sink organ [17,34]. Non-woven fabric containers significantly outperformed black plastic bags and rigid plastic pots across all shading levels [38]. The porous structure of non-woven fabric enables air root pruning, promoting lateral root proliferation and fibrous root systems, thereby enhancing water and nutrient uptake [31]. Similar improvements have been reported in *Araucaria angustifolia* (Bertol.) Steud. [41]. Yang et al. [33] showed in *Castanopsis hystrix* Hook. f. & Thomson ex A. DC. that non-woven containers increase fine root density and induce divergent root distribution, whereas plastic containers cause root coiling; such deformities persist after outplanting and may compromise stability [37]. In our study, black plastic bags, which are less permeable, had the highest POD activity yet also the highest MDA content (except at 20% shading). This pattern is consistent with the positive correlation between POD activity and MDA content, suggesting that their antioxidant system was activated but may have been insufficient to prevent oxidative damage, possibly due to poor aeration and large temperature fluctuations exacerbating ROS production beyond scavenging capacity [52]. Seedlings in black plastic bags also had the lowest root dry weight and root-to-shoot ratio across all shading levels, indicating compromised root biomass. In contrast, non-woven containers are likely to maintain better root-zone aeration, supporting aerobic respiration, nutrient uptake, and better root development [32,41]. Collectively, container type influenced seedling performance through both physical root restriction and root-zone environmental differences, although these interpretations remain correlational because rhizosphere parameters were not directly measured.

Container effects interacted significantly with shading. Non-woven containers showed the greatest advantage under 0% and 20% shading, whereas under 60% and 80% shading, rigid plastic pots outperformed non-woven containers in height increment and Tchl. This reversal cannot be explained by container volume, as all containers were filled with the same volume of substrate. One speculative possibility is that under extreme low-light stress, root-zone aeration becomes less critical, and the thermal inertia or structural properties of rigid plastic pots may become advantageous [35,58,59]. Hormone signaling may also be involved: air-permeable containers could promote root cytokinin synthesis, enhancing stomatal opening and photosynthesis, whereas non-air-permeable containers might induce ABA accumulation, aggravating stomatal limitation [60]. Because we did not measure hormones or root-zone temperature, these interpretations are speculative. Similar interactions have been reported in *Ilex paraguariensis* A.St.-Hil. [40]. Another intriguing pattern is the U-shaped height response of non-woven containers, which decreased to a minimum at 60% shading and then increased at 80%, suggesting that non-woven containers may buffer early shading stress and sustain some growth under severe carbon limitation [40]. This interpretation, however, requires further investigation.

The PCA integrated 14 indicators and showed that the first two components explained 87.80% of the variance: PC1 (75.20%) represented productivity, and PC2 (12.60%) represented stress resistance, a growth–defense continuum consistent with Monson et al. [52] and validated in similar evaluations [32,61]. The full light + non-woven fabric combination achieved the highest score, followed by 20% shading + non-woven container and full light + rigid plastic pot, confirming that *D. odorifera* should be cultivated under full light [28] and that non-woven containers effectively integrate productivity and stress resistance [38]. Black plastic bags performed worst and should be avoided, consistent with observations in *Camphora bodinieri* (H. Lév.) Y. Yang, Bing Liu & Zhi Yang [62] and *Mytilaria laosensis* Lecomte [34]. From an ecological niche perspective, this ranking reflects a trade-off between the species’ strong light requirement and its plasticity to container type [9,28]. As a keystone species in tropical monsoon forests, *D. odorifera* has evolved deep adaptation to high light; its photosynthetic apparatus lacks efficient low-light regulatory mechanisms, leading to excess excitation energy under shade [55]. Consequently, shading triggers severe carbon limitation, and container type can only partially mitigate this. We therefore recommend non-woven fabric containers under full sunlight for optimal seedling quality. For high-temperature regions, 20% shading with non-woven containers may be an alternative, as mild shading can reduce heat stress and photoinhibition and potentially improve light use efficiency [12,13,17,18,19], although this was not directly measured. Non-woven containers remain intact throughout the nursery period, provide better root-ball integrity at transplanting, and their benefits outweigh the slight cost increase over black plastic bags [33,38]. However, because *D. odorifera* is strongly photophilous, full sunlight remains optimal under the tested conditions [28]. Several limitations should be acknowledged: the six-month nursery-stage experiment left post-planting performance and long-term root deformations unverified, and whether these nursery-stage effects persist or can be reversed after outplanting remains unknown; rhizosphere conditions were not directly quantified, and root morphological traits were not assessed, so mechanistic chains remain correlational; antioxidant assays captured only terminal responses, and the proposed hormonal mechanisms and U-shaped height response require direct validation. Future research should integrate in situ rhizosphere monitoring, long-term field trials, transcriptomic and metabolomic approaches, root-zone hormone measurements, root imaging, and intervention experiments (e.g., altering container air permeability) to test causality.

## 4. Materials and Methods

### 4.1. Site Area

The experiment was conducted in 2024 at the nursery base of the Hainan Academy of Forestry (Hainan Academy of Mangrove) (19°52′ N, 110°28′ E). The site has an elevation of 30.4 m. The region experiences a tropical monsoon marine climate, with a mean annual temperature of 23.8 °C, annual precipitation of 1697.8 mm, and annual sunshine duration of 1750–2000 h. The natural soil at the site is lateritic red soil. For container cultivation, the growth substrate was a mixture of red soil, peat, and perlite (2:1:1, *v*/*v*/*v*), with initial properties as reported by [5]: pH 4.9, total N 6.82 g/kg, total P 0.69 g/kg, and total K 3.42 g/kg.

### 4.2. Materials and Experimental Design

Two-year-old *Dalbergia odorifera* T. Chen seedlings propagated from seed were used. Healthy individuals with uniform growth were selected, with an initial height of 56.75 ± 7.88 cm and basal diameter of 6.56 ± 0.96 mm.

To investigate the interactive effects of shading intensity and container type on seedling performance, a two-factor completely randomized design was established in June 2024 and concluded in December 2024, covering the main growing season of *D. odorifera* in Hainan. This 6-month period spans the active growth phase from seedling establishment to the onset of winter dormancy, which is the typical nursery production cycle for this species before outplanting. In local practice, seedlings are normally transplanted to the field in the following spring (March–April), after approximately 6 months of nursery cultivation [7,28]. The first factor, shading intensity, comprised five levels achieved using black polyethylene shade nets (Lvandi brand, Greenland Shade Company, Taizhou, China) installed 2.0 m above ground: 0% (S1), 20% (S2), 40% (S3), 60% (S4), and 80% (S5) shading (corresponding to 100%, 80%, 60%, 40%, and 20% of full sunlight, respectively). These shading levels were selected based on previous nursery management practices and covered a gradient ranging from full sunlight to severe shade conditions [28]. Each shading level was independently replicated in three separate shade structures, with shade nets covering the entire structure and extending at least 0.5 m beyond the edges to minimize side-light interference. The second factor included three container types commonly used in nursery production (MiaoFule brand, MiaoFule Horticulture Technology Co., Ltd., Anqing, China): rigid plastic pots (C1), non-woven fabric bags (C2), and black plastic seedling bags (C3). These three container types represent the most commonly used nursery containers in southern China and differ primarily in their aeration capacity and root-guiding properties [37]. To ensure that treatment effects were attributable to container material rather than volume, containers with comparable capacities were selected: C1 (top diameter 30 cm × height 25 cm), C2 (diameter 30 cm × height 30 cm), and C3 (diameter 28 cm × height 35 cm). All containers were filled with the same volume of growth substrate (21 L) to ensure comparability. The volume variation among the three container types was below 5%, which is unlikely to bias treatment comparisons.

The experiment was arranged as follows. Shading treatments were randomly assigned to independent shade structures. Within each shade structure, the three container types were randomly assigned to three independent plots, giving a total of 15 treatment combinations (5 shading levels × 3 container types). Each treatment combination was replicated three times, corresponding to the three independent shade structures per shading level. Each plot contained five seedlings, with one seedling per container, and the five seedlings were treated as observational units. The plot (i.e., the shading × container combination) was the experimental unit for statistical analysis (*n* = 3 per treatment), and all statistical analyses were performed on plot means to avoid pseudoreplication. Seedlings were spaced 50 cm apart within each plot to minimize mutual shading. Plots were separated by 0.5–1.0 m buffer zones to reduce edge effects and interference between adjacent treatments. Photosynthetically active radiation (PAR) transmittance for each shading level was calibrated at the start of the experiment using a quantum sensor (LI-COR Biosciences, Lincoln, NE, USA) to confirm target intensities. Throughout the experimental period, standardized drip irrigation maintained soil moisture at 60–70% of field capacity, and light transmission was monitored biweekly to ensure shade net integrity and treatment fidelity.

### 4.3. Measurement Parameters

(1) Growth parameters

At the beginning and end of the experiment, plant height and basal diameter were measured for all seedlings in each treatment. Plant height was determined using a steel tape measure (8208, Deli Group Co., Ltd., Ningbo, China) from the substrate surface to the base of the terminal bud. Basal diameter was measured using a digital vernier caliper (SHG-200MM, Shanghai Tool Works Co., Ltd., Shanghai, China) at 1 cm above the substrate surface. Plant height increment and basal diameter increment were subsequently calculated. Upon completion of the experiment, three representative seedlings per replicate were selected from each treatment and separated into root, stem, and leaf fractions. Tissue samples were oven-dried at 105 °C for 30 min to rapidly deactivate enzymes and prevent respiration loss, then at 80 °C to constant weight to avoid tissue combustion during prolonged drying [28]. Dry weights of roots, stems, and leaves were recorded using an electronic balance (Sartorius AG, Göttingen, Germany), and total dry biomass was computed by summation. Based on these measurements, height-to-diameter ratio, root-to-shoot ratio, and Dickson quality index were calculated The Dickson quality index (DQI) was calculated as: DQI = total dry weight (g)/[(height-to-diameter ratio) + (stem dry weight/root dry weight)] [26].

(2) Photosynthetic characterization

Photosynthetic measurements were conducted on clear mornings between 9:00 and 11:00 in mid-October 2024. For each treatment, four seedlings (selected across the three replicates) with representative growth, free from visible damage or disease and receiving adequate light exposure, were selected. From each plant, the third fully expanded mature leaf below the apical bud was sampled. This leaf position was selected because it represents a mature, fully functional leaf that has completed expansion and is actively photosynthesizing, minimizing age-related variability in photosynthetic capacity. Net photosynthetic rate (*P*_n_), intercellular CO_2_ concentration (*C*_i_), stomatal conductance (*g*_s_), and transpiration rate (*T*_r_) were measured using a Li-6400 portable photosynthesis system (LI-COR Biosciences, Lincoln, NE, USA). The measurement conditions were set as follows: 500 μmol/s airflow, 400 μmol/mol reference CO_2_ level, 25 °C leaf chamber temperature, 60% relative humidity, and 10,000 rpm cooling fan speed. The units for the measured parameters were: *P*_n_ in μmol CO_2_ m^−2^ s^−1^, *C*_i_ in μmol CO_2_ mol^−1^, *g*_s_ in mol H_2_O m^−2^ s^−1^, and *T*_r_ in mmol H_2_O m^−2^ s^−1^. Three leaves per plant were measured, and the average value per treatment was calculated from all measured leaves (4 plants × 3 leaves = 12 measurements per treatment) [29].

Chlorophyll content was determined by propanol extraction [50]. Fresh leaves (0.5 g, main vein excised) were finely chopped and homogenized with 5 mL propanol (Solarbio Science & Technology Co., Ltd., Beijing, China) and quartz sand (Solarbio Science & Technology Co., Ltd., Beijing, China) under ice-cold conditions. The homogenate was transferred to a centrifuge tube, diluted to 10 mL with propanol, and incubated at 4 °C in darkness for 24 h with intermittent agitation. Following centrifugation (4000× *g*, 10 min), supernatant absorbance was measured at 663 nm (chlorophyll a) and 645 nm (chlorophyll b) against a propanol blank using a spectrophotometer (UV-1050, Shanghai Techcomp Scientific Instrument Co., Ltd., Shanghai, China). Chlorophyll concentrations were calculated according to Arnon’s equations:

Chlorophyll a (mg/g) = [(12.7 × *A*_663_) − (2.69 × *A*_645_)] × *V*/(1000 × *W*)

Chlorophyll b (mg/g) = [(22.9 × *A*_645_) − (4.68 × *A*_663_)] × *V*/(1000 × *W*)

Total Chlorophyll (Tchl) = Chlorophyll a + Chlorophyll b

where *A* denotes absorbance, *V* is extract volume (mL), and *W* is sample fresh weight (g).

(3) Leaf physiological parameters

At the conclusion of the experiment, functional leaves (fully expanded, healthy leaves from the middle canopy) were collected from each treatment (three leaves per seedling). Samples were immediately frozen in liquid nitrogen and stored at −80 °C for subsequent physiological analyses.

Antioxidant enzyme activities and membrane lipid peroxidation products were determined using commercial assay kits (Solarbio Science & Technology Co., Ltd., Beijing, China) according to the manufacturer’s instructions. Briefly, frozen leaf samples (0.1 g FW) were homogenized in 1 mL of ice-cold extraction buffer (50 mM phosphate buffer, pH 7.8, containing 1 mM EDTA and 0.1% (*v*/*v*) Triton X-100; Solarbio Science & Technology Co., Ltd., Beijing, China) and centrifuged at 12,000× *g* for 15 min at 4 °C. The supernatant was used for all assays.

Superoxide dismutase (SOD) activity was assayed using the nitroblue tetrazolium (NBT) photoreduction method. One unit of SOD activity was defined as the amount of enzyme required to inhibit 50% of NBT photoreduction at 560 nm, and activity was expressed as U g^−1^ FW. Peroxidase (POD) activity was measured by the guaiacol oxidation method. One unit of POD activity was defined as the amount of enzyme that caused a change of 0.01 in absorbance at 470 nm per minute, and activity was expressed as U g^−1^ FW. Proline (PRO) content was quantified using the acid-ninhydrin method. Absorbance was measured at 520 nm, and PRO content was calculated from a standard curve using L-proline (Solarbio Science & Technology Co., Ltd., Beijing, China) and expressed as μg g^−1^ FW. Malondialdehyde (MDA) content was determined by the thiobarbituric acid (TBA) method. Absorbance was measured at 532 nm and corrected for non-specific absorbance at 600 nm. MDA content was calculated using the extinction coefficient of 155 mM^−1^ cm^−1^ and expressed as μmol g^−1^ FW [18,19].

### 4.4. Data Analysis

Raw data were organized using Microsoft Excel 2021 (Microsoft Corp., Redmond, WA, USA). All statistical analyses were performed using SPSS 26.0 (IBM Corp., Armonk, NY, USA). A two-way analysis of variance (ANOVA) was used to evaluate the main and interactive effects of shading intensity and container type on all measured variables. Prior to ANOVA, normality was checked using the Shapiro–Wilk test and homogeneity of variances was assessed using Levene’s test. All measured variables satisfied the assumptions of normality and homogeneity of variances (*p* > 0.05 for all tests); therefore, no data transformation was required. Duncan’s multiple range test was applied for post hoc comparisons at a significance level of α = 0.05. This test was chosen because it is widely accepted in plant science for separating treatment means when the number of comparisons is moderate (here, 5 shading levels × 3 container types = 15 combinations) and it provides effective control of the type I error rate. Pearson correlations were computed using plot-level means to assess relationships among growth, photosynthetic, and antioxidant indicators. Because shading may affect multiple traits simultaneously, these correlations should be interpreted as associations rather than causal relationships. Prior to principal component analysis (PCA), all variables were standardized using Z-score transformation to ensure equal weighting and prevent variables with larger numerical variance from dominating the analysis. PCA was performed on the 14 standardized growth and physiological indicators to reduce dimensionality (i.e., to condense the information from 14 correlated variables into a smaller number of uncorrelated components). The number of principal components to retain was determined based on eigenvalues > 1 and the cumulative proportion of variance explained. A comprehensive score for each treatment combination was subsequently calculated using the factor scores weighted by the proportion of variance explained by each retained principal component. All figures were generated using Origin 2022 (OriginLab Corp., Northampton, MA, USA). Data are presented as means ± standard error (SE).

## 5. Conclusions

This study demonstrates that shading intensity and container type synergistically regulate the growth, photosynthesis, and antioxidant systems of *Dalbergia odorifera* T. Chen seedlings. Shading-induced carbon limitation drives a carbon–antioxidant trade-off: moderate shading enhances antioxidant defense, whereas severe shading leads to oxidative damage. Container type significantly modulates this trade-off. Non-woven fabric containers improve root development and maintain higher photosynthetic efficiency under shade, whereas black plastic bags exacerbate stress. The combination of full sunlight and non-woven fabric containers showed the best performance among the tested treatments, yielding the highest overall seedling quality. Mild shading combined with non-woven containers offers a viable alternative for high-temperature regions. Practically, these findings provide guidance for nursery production of high-quality *D. odorifera* seedlings, a prerequisite for successful field establishment; optimizing light and container type during the nursery stage can thus support plantation establishment of this endangered species. Future research should integrate field trials to verify post-planting performance, along with rhizosphere monitoring and molecular approaches to further elucidate the signaling networks underlying light-root interactions.

## Figures and Tables

**Figure 1:**
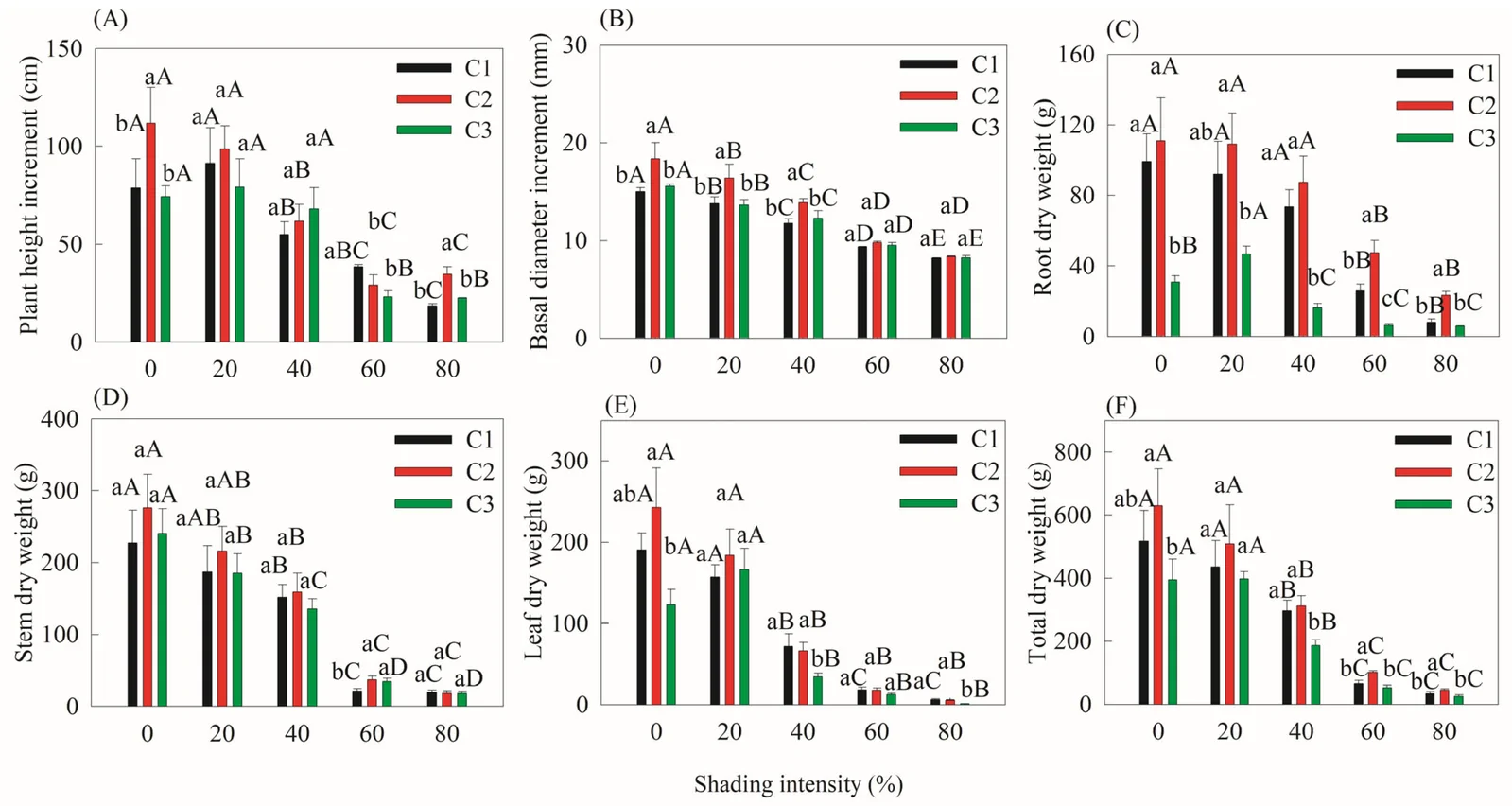
Effects of shading intensity and container type on growth parameters of *D. odorifera* seedlings. (**A**) Plant height increment; (**B**) Basal diameter increment; (**C**) Root dry weight; (**D**) Stem dry weight; (**E**) Leaf dry weight; (**F**) Total dry weight. Container types: C1, rigid plastic pots; C2, non-woven fabric bags; C3, black plastic seedling bags. Data are presented as means ± standard error (SE). Different lowercase letters indicate significant differences among container types within the same shading intensity, and different uppercase letters indicate significant differences among shading intensities within the same container type (Two-way ANOVA followed by Duncan’s multiple range test, *p* < 0.05).

**Figure 2:**
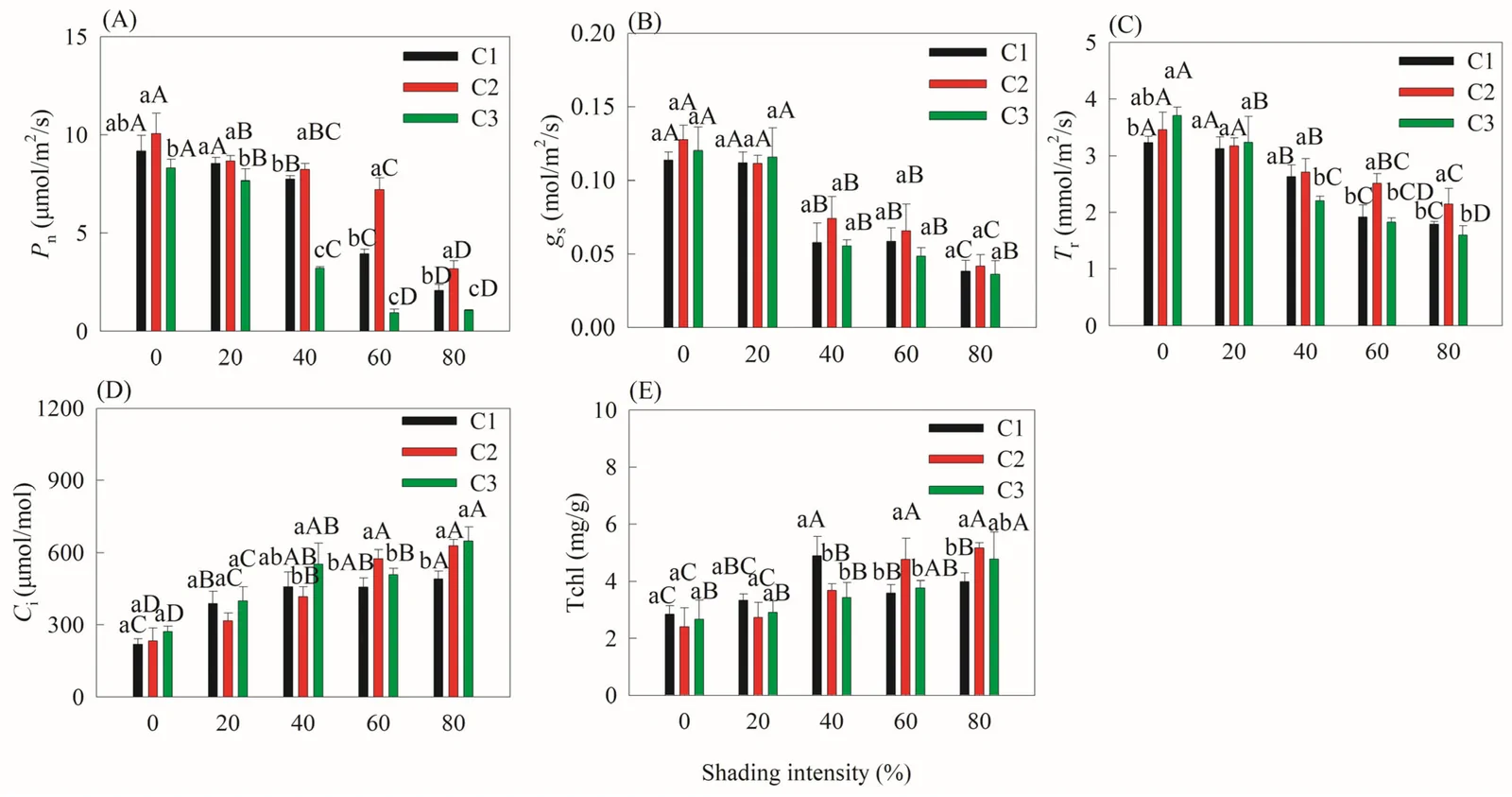
Effects of shading intensity and container type on photosynthetic parameters of *D. odorifera* seedlings. (**A**) Net photosynthetic rate (*P*_n_); (**B**) Stomatal conductance (*g*_s_); (**C**) Transpiration rate (*T*_r_); (**D**) Intercellular CO_2_ concentration (*C*_i_); (**E**) Total chlorophyll content (Tchl). Container types: C1, rigid plastic pots; C2, non-woven fabric bags; C3, black plastic seedling bags. Data are presented as means ± standard error (SE). Different lowercase letters indicate significant differences among container types within the same shading intensity, and different uppercase letters indicate significant differences among shading intensities within the same container type (Two-way ANOVA followed by Duncan’s multiple range test, *p* < 0.05).

**Figure 3:**
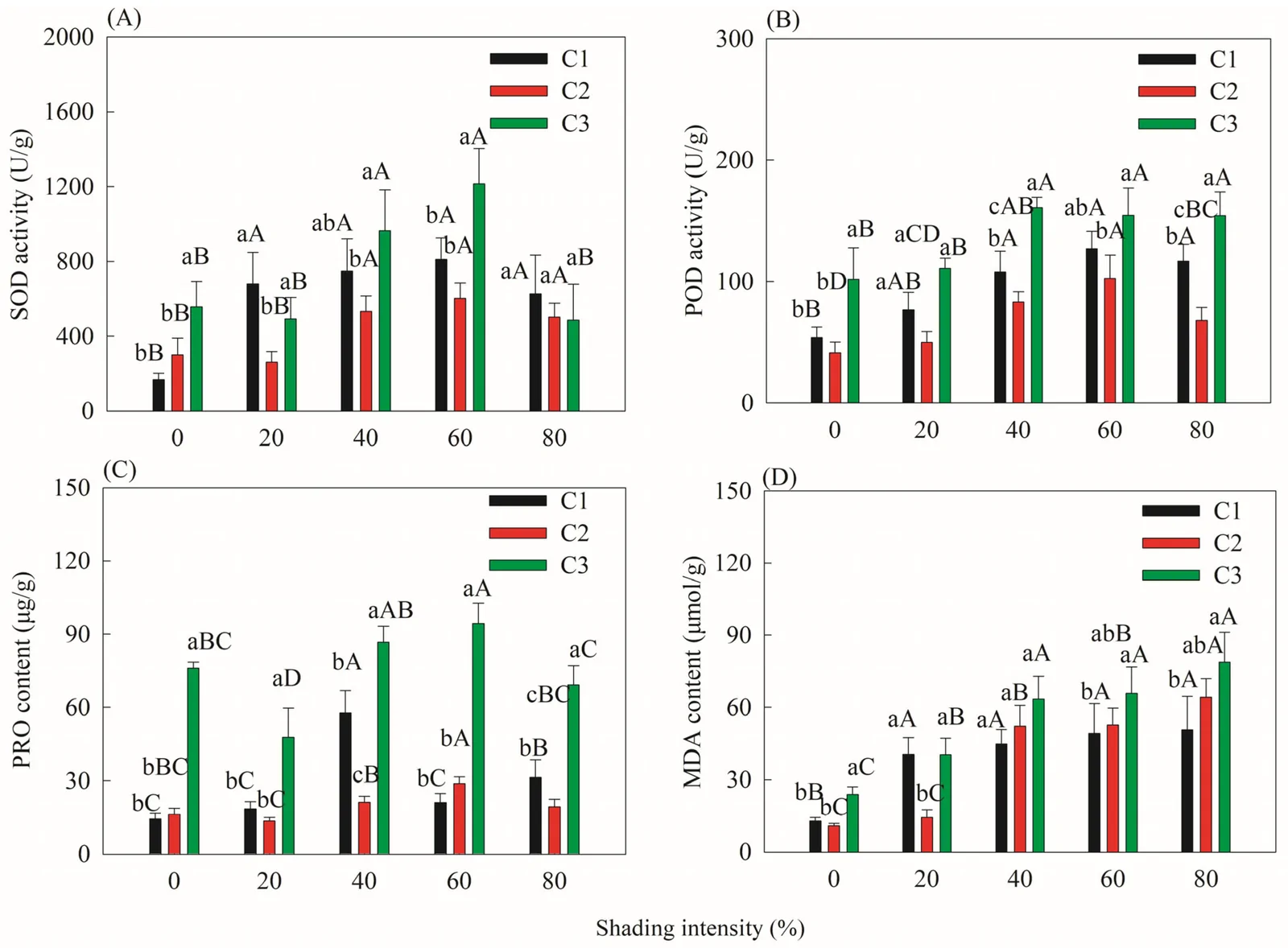
Effects of shading intensity and container type on antioxidant systems and membrane lipid peroxidation of *D. odorifera* seedlings. (**A**) Superoxide dismutase (SOD) activity; (**B**) Peroxidase (POD) activity; (**C**) Proline (PRO) content; (**D**) Malondialdehyde (MDA) content. Container types: C1, rigid plastic pots; C2, non-woven fabric bags; C3, black plastic seedling bags. Data are presented as means ± standard error (SE). Different lowercase letters indicate significant differences among container types within the same shading intensity, and different uppercase letters indicate significant differences among shading intensities within the same container type (Two-way ANOVA followed by Duncan’s multiple range test, *p* < 0.05).

**Figure 4:**
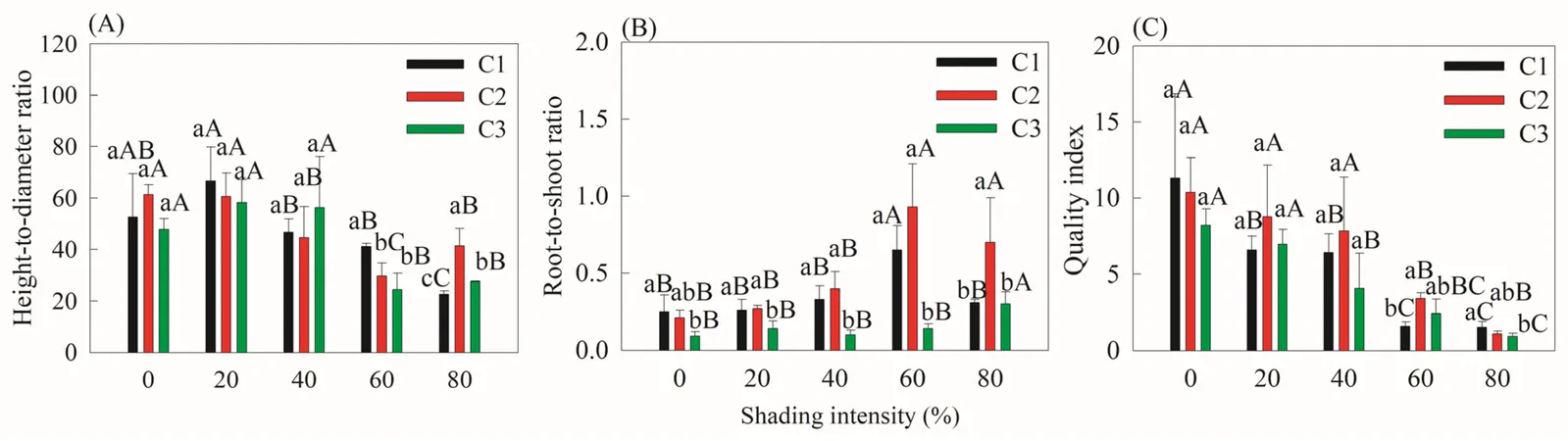
Effects of shading intensity and container type on morphological indices and quality of *D. odorifera* seedlings. (**A**) Height-to-diameter ratio; (**B**) Root-to-shoot ratio; (**C**) Dickson quality index. Container types: C1, rigid plastic pots; C2, non-woven fabric bags; C3, black plastic seedling bags. Data are presented as means ± standard error (SE). Different lowercase letters indicate significant differences among container types within the same shading intensity, and different uppercase letters indicate significant differences among shading intensities within the same container type (Two-way ANOVA followed by Duncan’s multiple range test, *p* < 0.05).

**Figure 5:**
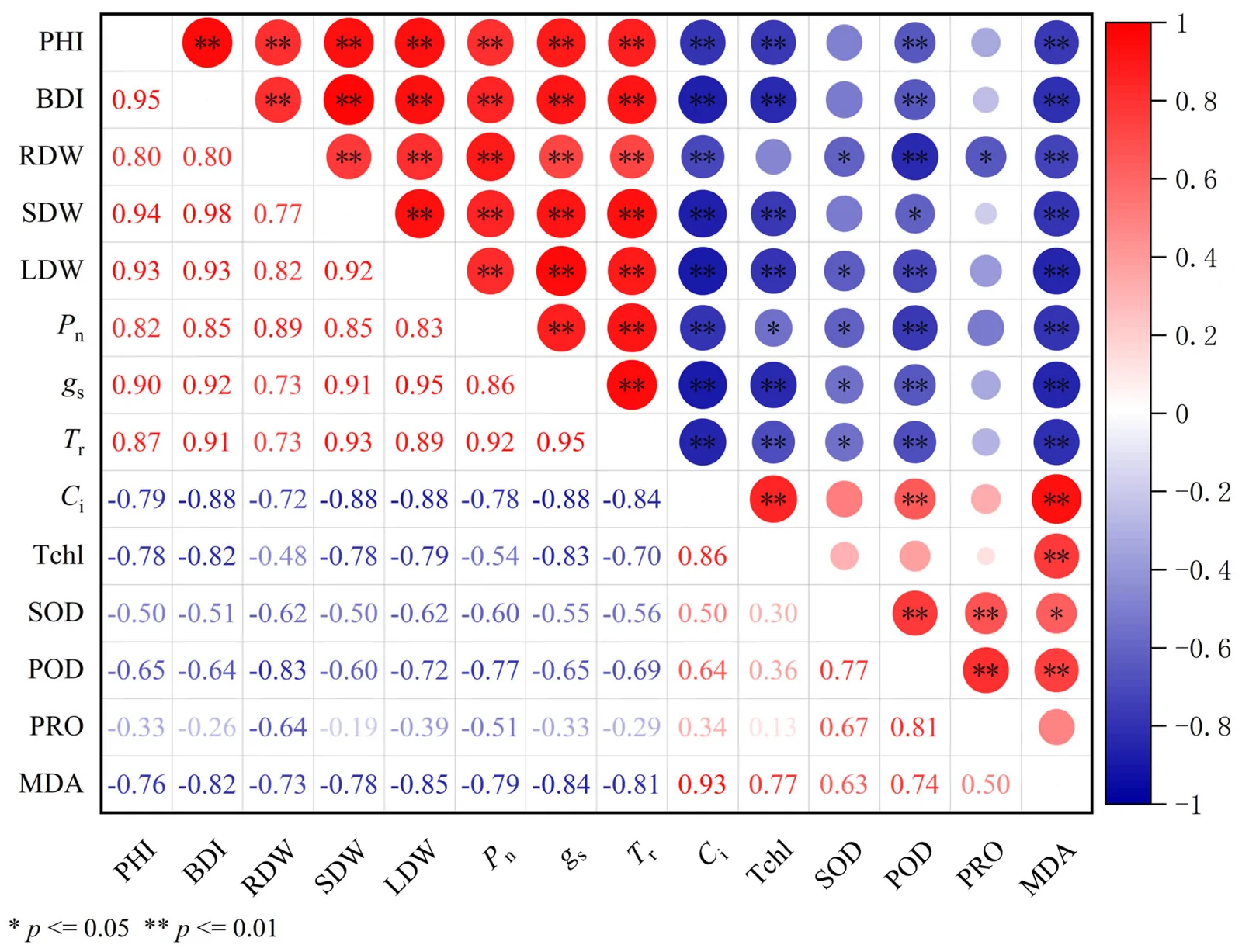
Heatmap of correlations among 14 growth, photosynthetic, and antioxidant indicators in *D. odorifera* seedlings under different shading intensities and container types. * indicates significant correlation (*p* < 0.05); ** indicates highly significant correlation (*p* < 0.01). Analyzed indicators include plant height increment (PHI), basal diameter increment (BDI), leaf dry weight (LDW), stem dry weight (SDW), root dry weight (RDW), net photosynthetic rate (*P*_n_), stomatal conductance (*g*_s_), transpiration rate (*T*_r_), intercellular CO_2_ concentration (*C*_i_), total chlorophyll content (Tchl), superoxide dismutase activity (SOD), peroxidase activity (POD), proline content (PRO), and malondialdehyde content (MDA).

**Figure 6:**
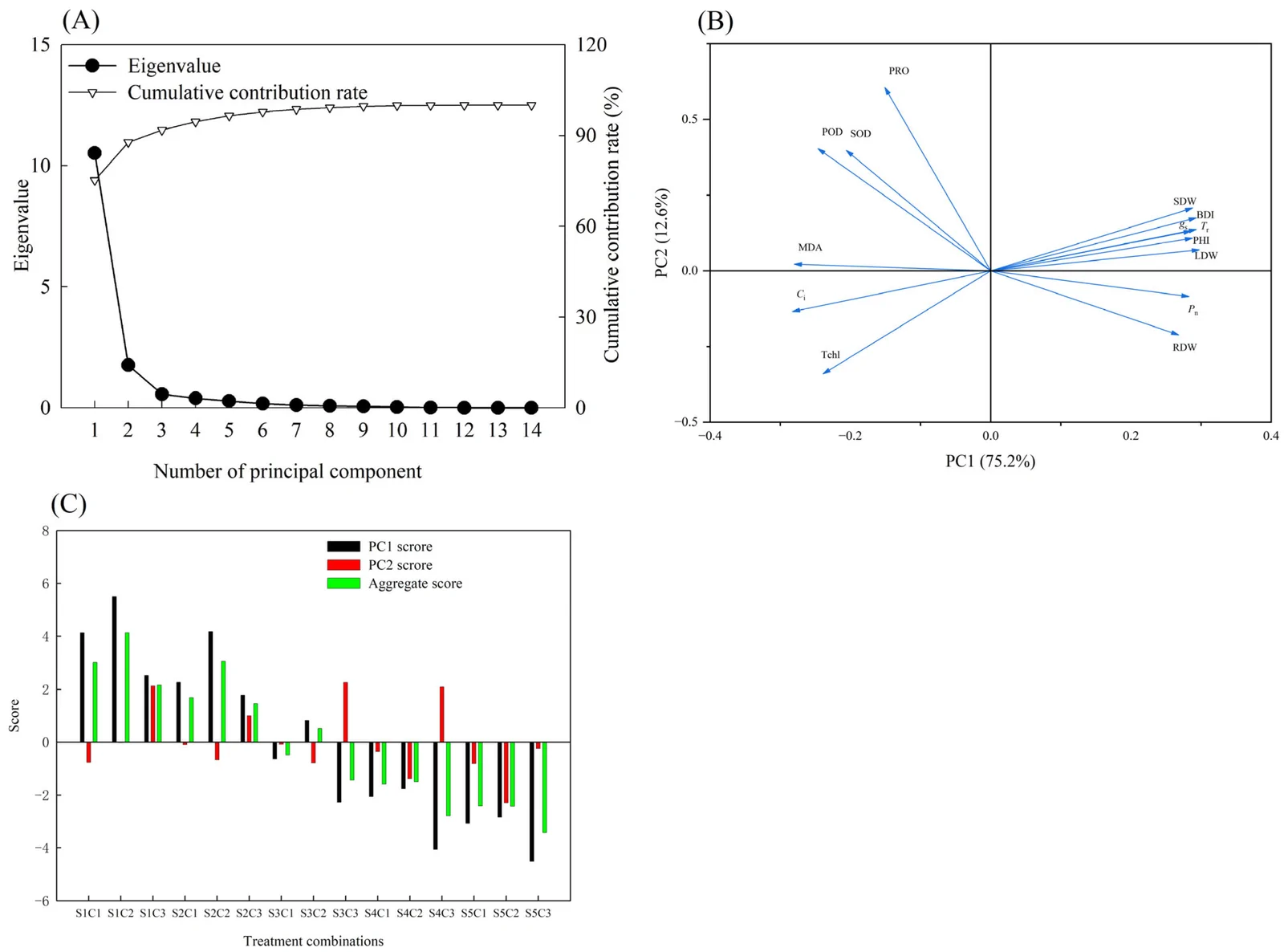
Principal component analysis (PCA) of growth and physiological indicators in *D. odorifera* seedlings under different shading intensities and container types. (**A**) Plot showing eigenvalues and cumulative variance explained by the principal components; (**B**) PCA loading plot of 14 growth and physiological indicators; (**C**) PCA score plot of treatment combinations. Shading intensities: S1, 0%; S2, 20%; S3, 40%; S4, 60%; S5, 80% full sunlight. Container types: C1, rigid plastic pots; C2, non-woven fabric bags; C3, black plastic seedling bags. Treatment combinations are labeled accordingly (e.g., S1C1: 0% shading + rigid plastic pots). Analyzed indicators include plant height increment (PHI), basal diameter increment (BDI), leaf dry weight (LDW), stem dry weight (SDW), root dry weight (RDW), net photosynthetic rate (*P*_n_), stomatal conductance (*g*_s_), transpiration rate (*T*_r_), intercellular CO_2_ concentration (*C*_i_), total chlorophyll content (Tchl), superoxide dismutase activity (SOD), peroxidase activity (POD), proline content (PRO), and malondialdehyde content (MDA).

**Table 1:** Two-factor ANOVA of the effects of shading degree and container type on seedling growth and biomass accumulation of *Dalbergia odorifera* T. C. Chen seedlings.

Parameters	Shading Intensity		Container Type		Shading Intensity × Container Type	
	*F* Value	*p* Value	*F* Value	*p* Value	*F* Value	*p* Value
Plant height increment (cm)	61.15	0.00 **	5.66	0.01 **	2.32	0.04 *
Basal diameter increment (mm)	223.56	0.00 **	29.40	0.00 **	4.06	0.00 **
Root dry weight (g)	24.30	0.00 **	31.76	0.00 **	1.88	0.09
Stem dry weight (g)	85.64	0.00 **	1.61	0.21	0.40	0.92
Leaf dry weight (g)	79.26	0.00 **	5.86	0.01 **	2.34	0.03 *
Total dry weight (g)	24.82	0.00 **	20.57	0.00 **	9.33	0.00 **

* indicates *p* < 0.05, ** indicates *p* < 0.01.

**Table 2:** Two-factor ANOVA of the effects of shading degree and container type on the photosynthetic physiological characteristics of *D. odorifera* seedlings.

Parameters	Shading Intensity		Container Type		Shading Intensity × Container Type	
	*F* Value	*p* Value	*F* Value	*p* Value	*F* Value	*p* Value
*P*_n_ (μmol/m^2^/s)	358.22	0.00 **	185.39	0.00 **	21.84	0.00 **
*g*_s_ (mol/m^2^/s)	89.25	0.00 **	2.49	0.09	0.76	0.64
*T*_r_ (mmol/m^2^/s)	91.87	0.00 **	7.93	0.00 **	3.60	0.00 **
*C*_i_ (μmol/mol)	74.75	0.00 **	8.98	0.00 **	3.98	0.00 **
Tchl (mg/g)	23.20	0.00 **	1.00	0.37	4.08	0.00 **

** indicates *p* < 0.01.

**Table 3:** Two-way ANOVA results for the effects of shading intensity and container type on antioxidant systems and membrane lipid peroxidation in *D. odorifera* seedlings.

Parameters	Shading Intensity		Container Type		Shading Intensity × Container Type	
	*F* Value	*p* Value	*F* Value	*p* Value	*F* Value	*p* Value
SOD activity (U/g)	18.15	0.00 **	15.13	0.00 **	3.84	0.00 **
POD activity (U/g)	15.75	0.00 **	42.32	0.00 **	0.59	0.79
PRO content (μg/g)	48.10	0.00 **	16.70	0.00 **	4.31	0.00 **
MDA content (μmol/g)	31.40	0.00 **	371.70	0.00 **	12.44	0.00 **

** indicates *p* < 0.01.

**Table 4:** Two-way ANOVA results for the effects of shading intensity and container type on seedling morphological structure and quality index of *D. odorifera* seedlings.

Parameters	Shading Intensity		Container Type		Shading Intensity × Container Type	
	*F* Value	*p* Value	*F* Value	*p* Value	*F* Value	*p* Value
Height-to-diameter ratio	23.761	0.00 **	1.82	0.17	3.25	0.00 **
Root-to-shoot ratio	24.82	0.00 **	20.57	0.00 **	9.33	0.00 **
Dickson quality index	24.83	0.00 **	2.54	0.09	0.71	0.68

** indicates *p* < 0.01.

## Data Availability

The original contributions presented in this study are included in the article. Further inquiries can be directed to the corresponding author.

## Appendix A

**Table A1:** Means ± SD of growth and biomass parameters for the 15 shading × container combinations.

Treatment	Plant Height Increment (cm)	Basal Diameter Increment (mm)	Root Dry Weight (g)	Stem Dry Weight (g)	Leaf Dry Weight (g)	Total Dry Weight (g)
S1C1	78.64 ± 14.92	14.99 ± 0.43	99.21 ± 15.67	227.51 ± 45.49	190.85 ± 20.67	517.57 ± 97.00
S1C2	111.92 ± 18.09	18.34 ± 1.68	111.04 ± 24.09	276.16 ± 46.80	242.97 ± 48.42	630.17 ± 116.00
S1C3	74.35 ± 5.53	15.55 ± 0.23	30.79 ± 3.69	240.84 ± 34.22	123.29 ± 18.65	394.93 ± 65.51
S2C1	91.41 ± 18.04	13.79 ± 0.71	92.11 ± 18.45	187.20 ± 36.65	157.06 ± 15.56	436.36 ± 83.84
S2C2	98.72 ± 11.66	16.41 ± 1.40	109.07 ± 17.68	216.00 ± 33.91	184.11 ± 31.98	509.18 ± 123.79
S2C3	79.22 ± 14.42	13.66 ± 0.55	46.71 ± 4.55	185.26 ± 26.86	166.49 ± 25.95	398.46 ± 23.06
S3C1	54.96 ± 6.48	11.76 ± 0.45	73.56 ± 9.55	151.98 ± 17.55	71.68 ± 15.85	297.22 ± 32.95
S3C2	61.81 ± 8.59	13.89 ± 0.41	87.41 ± 14.70	158.87 ± 26.45	66.25 ± 10.66	312.53 ± 32.42
S3C3	68.01 ± 10.91	12.28 ± 0.76	16.28 ± 2.50	135.52 ± 14.48	34.51 ± 4.60	186.31 ± 19.20
S4C1	38.45 ± 1.14	9.35 ± 0.04	25.98 ± 3.58	21.65 ± 3.21	18.35 ± 3.77	65.98 ± 10.55
S4C2	29.02 ± 5.26	9.78 ± 0.13	47.49 ± 6.95	36.98 ± 5.11	17.97 ± 3.02	102.43 ± 4.95
S4C3	23.26 ± 2.96	9.53 ± 0.25	6.43 ± 0.83	34.79 ± 4.42	12.35 ± 1.62	53.57 ± 7.59
S5C1	18.58 ± 1.05	8.22 ± 0.02	8.19 ± 1.81	19.80 ± 2.98	6.20 ± 1.29	34.19 ± 7.12
S5C2	34.70 ± 3.75	8.38 ± 0.05	23.30 ± 2.35	18.03 ± 3.91	5.54 ± 0.50	45.09 ± 4.11
S5C3	22.63 ± 0.11	8.25 ± 0.23	5.86 ± 0.28	18.30 ± 2.90	1.16 ± 0.01	25.32 ± 5.21

**Table A2:** Means ± SD of photosynthetic and leaf physiological parameters for the 15 shading × container combinations.

Treatment	*P*_n_ (μmol/m^2^/s)	*g*_s_ (mol/m^2^/s)	*T*_r_ (mmol/m^2^/s)	*C*_i_ (μmol/mol)	Tchl (mg/g)	SOD Activity (U/g)	POD Activity (U/g)	PRO Content (μg/g)	MDA Content (μmol/g)
S1C1	9.17 ± 0.80	0.114 ± 0.006	3.23 ± 0.11	219.80 ± 21.45	2.84 ± 0.29	168.62 ± 31.23	53.75 ± 8.73	14.44 ± 2.24	12.98 ± 1.49
S1C2	10.05 ± 1.04	0.128 ± 0.010	3.46 ± 0.31	232.10 ± 54.49	2.40 ± 0.66	299.53 ± 89.70	41.27 ± 8.95	16.23 ± 2.46	10.92 ± 1.03
S1C3	8.32 ± 0.45	0.120 ± 0.016	3.71 ± 0.15	270.77 ± 23.01	2.66 ± 0.67	555.32 ± 137.53	101.98 ± 25.81	76.06 ± 2.48	23.85 ± 3.29
S2C1	8.55 ± 0.30	0.112 ± 0.007	3.12 ± 0.21	388.12 ± 51.39	3.32 ± 0.23	680.51 ± 168.12	76.62 ± 14.37	18.37 ± 3.10	40.58 ± 6.83
S2C2	8.68 ± 0.28	0.112 ± 0.006	3.17 ± 0.14	316.34 ± 33.05	2.73 ± 0.53	261.50 ± 55.44	49.70 ± 8.99	13.54 ± 1.60	14.48 ± 3.05
S2C3	7.66 ± 0.61	0.116 ± 0.020	3.23 ± 0.46	397.99 ± 60.21	2.91 ± 0.39	493.25 ± 113.99	110.98 ± 8.30	47.68 ± 11.89	40.46 ± 6.63
S3C1	7.75 ± 0.17	0.058 ± 0.013	2.63 ± 0.21	458.06 ± 60.78	4.89 ± 0.68	748.76 ± 170.83	107.76 ± 17.28	57.63 ± 9.22	44.71 ± 6.02
S3C2	8.24 ± 0.31	0.074 ± 0.015	2.71 ± 0.25	416.34 ± 42.88	3.68 ± 0.23	534.33 ± 80.41	83.20 ± 8.31	21.23 ± 2.38	52.15 ± 8.60
S3C3	3.21 ± 0.09	0.056 ± 0.004	2.20 ± 0.08	551.42 ± 87.32	3.42 ± 0.53	963.14 ± 219.70	160.95 ± 8.29	86.68 ± 6.54	63.44 ± 9.43
S4C1	3.95 ± 0.24	0.058 ± 0.009	1.92 ± 0.21	457.01 ± 36.77	3.58 ± 0.30	811.54 ± 112.57	127.02 ± 14.31	21.03 ± 3.70	49.14 ± 12.43
S4C2	7.22 ± 0.58	0.066 ± 0.018	2.51 ± 0.17	575.00 ± 37.07	4.77 ± 0.74	601.67 ± 82.42	102.53 ± 19.18	28.92 ± 2.65	52.66 ± 7.00
S4C3	0.95 ± 0.19	0.048 ± 0.006	1.83 ± 0.07	507.29 ± 27.87	3.76 ± 0.26	1215.79 ± 187.95	154.70 ± 22.45	94.34 ± 8.31	65.81 ± 11.05
S5C1	2.09 ± 0.30	0.038 ± 0.007	1.79 ± 0.04	490.21 ± 32.72	3.98 ± 0.31	625.13 ± 209.31	116.96 ± 13.85	31.36 ± 7.28	50.65 ± 13.87
S5C2	3.18 ± 0.41	0.042 ± 0.008	2.14 ± 0.28	627.36 ± 26.76	5.16 ± 0.18	502.46 ± 73.60	67.98 ± 10.72	19.30 ± 3.09	64.13 ± 7.81
S5C3	1.07 ± 0.04	0.036 ± 0.009	1.60 ± 0.17	648.36 ± 58.67	4.78 ± 0.95	486.48 ± 190.85	154.47 ± 19.29	69.24 ± 7.83	78.81 ± 12.33

**Table A3:** Means ± SD of morphological and quality indices for the 15 shading × container combinations.

Treatment	Height-to-Diameter Ratio	Root-to-Shoot Ratio	Dickson Quality Index
S1C1	52.57 ± 16.89	0.25 ± 0.11	11.32 ± 5.52
S1C2	61.27 ± 3.92	0.21 ± 0.05	10.37 ± 2.29
S1C3	47.86 ± 4.06	0.09 ± 0.03	8.23 ± 1.07
S2C1	66.68 ± 13.11	0.26 ± 0.07	6.60 ± 0.92
S2C2	60.64 ± 9.13	0.27 ± 0.02	8.78 ± 3.40
S2C3	58.23 ± 8.83	0.14 ± 0.05	6.96 ± 0.98
S3C1	46.71 ± 5.08	0.33 ± 0.09	6.43 ± 1.23
S3C2	44.58 ± 12.04	0.40 ± 0.11	7.86 ± 3.52
S3C3	56.28 ± 19.83	0.10 ± 0.03	4.08 ± 2.30
S4C1	41.11 ± 1.34	0.65 ± 0.16	1.59 ± 0.29
S4C2	29.62 ± 5.10	0.93 ± 0.28	3.42 ± 0.38
S4C3	24.43 ± 6.34	0.14 ± 0.03	2.41 ± 0.97
S5C1	22.61 ± 1.31	0.31 ± 0.02	1.51 ± 0.38
S5C2	41.40 ± 6.74	0.70 ± 0.29	1.09 ± 0.18
S5C3	27.44 ± 0.19	0.30 ± 0.08	0.91 ± 0.19
